# Why Do Some People Drink Too Much? The Role of Genetic and Psychosocial Influences

**Published:** 2000

**Keywords:** genetic theory of AODU (AOD [alcohol or other drug] use, abuse, and dependence), genetic trait, QTL mapping, genetic linkage, twin study, family AODU history, AOD sensitivity, protective factors, psychological development, attitude toward AOD

## Abstract

A sizable minority of the population abuses alcohol. Research has found that some vulnerability to developing alcohol-related problems is conveyed genetically, and animal research has indicated that inheritance can take many forms. Studies in mice have demonstrated that various individual genes or groups of genes can shape very distinct responses to alcohol. By identifying the proteins these genes encode and the mechanisms by which the genes influence an animal’s biochemical response to alcohol, scientists can gain insight into the features of human alcoholism and provide a basis for developing pharmaceuticals that short-circuit these genetically defined processes.

Alcohol is available to any adult, and illegally to many minors, in the United States. Although most people abstain or drink safely, a sizable minority of the population abuses alcohol. Understanding why alcohol leads to trouble for many, but not most, people exposed to it is the goal of research on the cause (i.e., etiology) of alcohol abuse and alcoholism. It is already clear that some vulnerability to developing alcohol-related problems is conveyed genetically, and animal research has indicated that inheritance can take many forms. Studies in mice have demonstrated that various individual genes or groups of genes can shape very distinct responses to alcohol, such as a preference for alcohol over water, sensitivity to alcohol’s intoxicating effects, and the tendency to develop tolerance to it. By identifying the proteins these genes encode and the mechanisms by which the genes influence an animal’s biochemical response to alcohol, scientists can gain insight into the features of human alcoholism and provide a basis for developing pharmaceuticals that short-circuit these genetically defined processes.

Another goal of research on the genetics of alcoholism in humans is to determine to what extent individual differences in alcohol-related behavior are due to genetic versus environmental influences. For example, in twin studies as much as two-thirds of the variability in drinking behavior in one population could be attributed to genetic factors in both men and women. Other twin studies are investigating the relative magnitude of various influences on alcohol consumption in youth. Identifying the genes that convey risk of alcoholism is a second major goal of genetic research; scans of the human genome reveal evidence of genes in certain chromosomal regions that influence alcoholism.

Understanding how inborn vulnerability plays out in the temperament and behavior of an individual and in the context of family, peers, and culture is the goal of psychosocial research on the cause of alcoholism. The traits and family characteristics of children at risk because of a family history of alcoholism also predict risk in children of nonalcoholic parents. If alcoholism represents the end result of a sequence to which many factors contributed, then the hope is that by understanding the contributors and how they interact, it also will be possible to intervene before vulnerability becomes a destructive illness.

## Animal Genetic Studies on Alcoholism

Vulnerability to alcohol dependence and abuse is partly determined by genes. However, no single gene is responsible for alcohol abuse and dependence; rather, many genes that play roles in a variety of normal human behaviors and sensory perception are involved. Identifying all the genes involved is a project of enormous magnitude and difficulty, because of the size of the human genome^1^ and the complexity of the behaviors involved in abusive drinking and dependence. The Human Genome Project (HGP) (supported in the United States by the National Institutes of Health and the U.S. Department of Energy) has been an important impetus to the search for genes involved in alcohol-related behaviors. HGP researchers are working toward the goal of identifying every gene and the protein it encodes and mapping each gene to a precise location (i.e., locus) on one of the chromosomes.^2^ This research is providing the tools with which scientists can investigate the genetic underpinnings of a range of human disorders and conditions, including alcohol abuse and dependence. Even with these advances in understanding the human genome, however, researchers continue to rely on animal models to investigate the causes and consequences of alcoholism.

As mentioned previously, vulnerability to alcohol dependence in humans and alcohol preference in animals are complex behaviors that are determined by multiple genes. Such traits are known as multigenic or quantitative traits. Rather than being simply present or absent, such traits are expressed along a spectrum from high to low. A technique developed in recent years for conducting the search for genes influencing such traits (i.e., quantitative trait loci [QTLs]) is called QTL mapping. This approach is based on the concept of linkage, which posits that genes located close together on the chromosome are more likely to be inherited together from one parent than are two genes farther apart. This technique provides a means of locating and measuring the effects of a single QTL on an observable trait or behavior (i.e., phenotype).

### Creating Rodent Models

Studies of animal genetics are useful because of fundamental limitations in human genetic studies. For example, the genetic blueprint of each human subject—except for those of identical twins—is unique, as are each person’s background and experiences. In contrast, laboratory researchers can control the mating of mice and rats over many generations and thereby produce strains of animals in which all individuals are genetically identical. Furthermore, researchers can control the environments of the animals. Because of the high degree of genetic similarity among individual animals and the extent of environmental control, researchers can attribute the differences in an alcohol-related behavior between two genetically dissimilar animal strains to differences in their genetic makeup. Moreover, because humans and rodents share most of their genes and because these genes produce proteins involved in identical physical processes in both species, the results of such animal genetic studies can provide insights into human genetics.

Animal genetics researchers use a variety of approaches to selectively breed mice and rats that display alcohol-related phenotypes similar to those of humans. Comparing the genetic makeup of different mouse strains should allow researchers to identify genetic underpinnings of the respective alcohol-related phenotypes.

Many researchers use mice from recombinant-inbred (RI) strains, especially mice from the BXD series, which contains 25 different strains. The series was created by crossing two mouse strains that are genetically distinct and differ from each other phenotypically in many ways, including many alcohol-related traits. Next, the researchers inbred many different pairs of offspring (brother-sister mating), which resulted in different strains of mice. Each mouse within a strain is genetically identical to every other mouse in that strain, but between any two of these strains only 50 percent of their genes are shared–the same amount that human siblings share. Thus, the different alcohol-related traits observed in the parents were sorted into individual animals and then fixed genetically. For example, RI mice from different strains can differ in their preferences for alcohol. The task for researchers then becomes to look for differences in the genetic makeup of these RI strains that might account for some of the differences in their alcohol preferences and to narrow the possible location of the responsible genes to specific chromosomal regions.

Steps Involved in QTL Mapping* in Rodents

1. Mate rodents (either recombinant inbred* mice or other types) that differ in genes involved in alcohol-related traits and behaviors.
2. Test individual offspring for the extent to which they display the characteristic or trait (i.e., phenotype).
3. Determine the pattern of genetic markers* in each of these mice.
4. Conduct statistical tests to determine whether any variation in the phenotype is significantly associated with any marker.
5. Perform additional statistical tests to determine the extent to which the marker affects the expression or predicts the variation of the multigenetic, or quantitative, trait.

*For a definition of this and other technical terms used in this article, see the glossary, p. 25.QTL = Quantitative trait loci.

### QTL Mapping

QTL mapping relies heavily on statistical analyses. These analyses are used to measure the degree of association between a marker and the phenotype under investigation to determine the magnitude of the effect of the QTL on the phenotype and to assess the statistical significance of the observed association between the marker(s) and the QTL. If the QTL is located close to the marker and has a large effect, then detection and mapping can be performed easily and accurately using simple statistical methods (e.g., regression analysis). If the QTL is farther away from the marker gene, a more complex and statistically optimal method called interval mapping can be used. Recently, researchers have developed still more sophisticated statistical methods, resulting in more accurate QTL mapping.

## Recent Studies of Alcohol-Related QTLs

In recent years, researchers have used the techniques described above to identify provisional QTLs for genes involved in several alcohol-related phenotypes exhibited by mice, including alcohol preference (which may reflect the rewarding properties that are closely related to alcohol’s addictive potential), sensitivity to alcohol’s sedative-hypnotic effects, and withdrawal. However, although many of these provisional QTLs will be subsequently confirmed by more refined studies, an unknown number will likely be found on further examination to be false positives. In addition, researchers are concerned about the potential for false negatives, or missing QTLs that are really there. Therefore, subsequent confirmation of provisional QTLs using other approaches is a statistical necessity.

One study screened the entire genome for major QTLs that might be involved in alcohol preference, identifying two loci, Alcp1 and Alcp2 (Melo et al. 1996). The two QTLs are gender specific, with Alcp1 being specific to males and Alcp2 being specific to females. Another series of studies focusing on sensitivity to alcohol’s sedative-hypnotic effects identified 12 provisional QTLs, out of which 2 or 3 were confirmed to be real. Finally, three QTLs for withdrawal have recently been confirmed (Buck et al. 1997). However, QTLs represent small chromosomal regions that can still contain numerous genes. No one has identified an individual gene responsible for differential alcohol sensitivity in rodent models.

### Shared Gene Actions

In other QTL mapping applications, investigators are interested in whether two distinct phenomena, such as sensitivity to alcohol’s effects and alcohol tolerance, result from the same underlying group of genes or from entirely separate QTLs. Because of the large number of diverse alcohol-related behaviors currently being investigated, analysis of potential shared gene actions is an important area for further work.

One study concluded that sensitivity and tolerance to alcohol are not mediated by common genetic factors (Phillips et al. 1996). In contrast, other researchers have presented evidence suggesting commonality in function between genes for sedative-hypnotic sensitivity to alcohol and genes specifying the distribution and levels of the brain chemical neurotensin, which plays a role in addiction (Erwin et al. 1997). Another study evaluating the relationship between sensitivity and tolerance found that most measures examined were not correlated, indicating that the traits had different genetic determinants (Crabbe et al. 1996). In general, there appear to be many more cases of different genes determining different measures of alcohol action, with relatively little commonality.

## Recent Progress in the Genetics of Alcoholism

Twin, family, and adoption studies have firmly established major roles for both genetics and environment in the etiology of alcoholism in humans. Such studies also have clearly demonstrated that alcoholism is a genetically complex disorder, influenced by multiple genes that interact in an unknown fashion with each other and with similarly unknown environmental factors to produce the disease. It also seems likely that individuals in different families develop alcoholism under the influence of different predisposing genes.

Researchers have identified two genes influencing predisposition to alcoholism. These genes encode certain forms of the enzymes alcohol dehydrogenase (ADH) and aldehyde dehydrogenase (ALDH), both of which are involved in alcohol metabolism. A defective variant (i.e., allele) of the ALDH2 gene, which is common in Asian populations, substantially (although not completely) protects carriers from developing alcoholism by causing a flushing reaction (e.g., facial flushing, nausea, vomiting, and a racing heart) after drinking alcohol. Newer studies have suggested that certain ADH2 and ADH3 alleles also protect carriers to some extent from developing alcoholism. These protective alleles, which are also common in Asian populations, ultimately act through a mechanism similar to the defective ALDH2 allele, producing feelings of discomfort and illness and thereby discouraging carriers of these alleles from consuming large amounts of alcohol.

## Findings From Twin/Family Studies

Classic twin studies compare the resemblances for a trait of interest between monozygotic (MZ, identical) twins and dizygotic (DZ, fraternal) twins in order to determine the extent of genetic influence, or heritability, of the trait. This comparison allows researchers to calculate heritability, because MZ twins are genetically identical, whereas DZ twins share only half their genes. The approach relies on the assumption, however, that for both MZ and DZ twins, environments of both individuals in the pair are equally similar. Recent studies also have collected environmental data to allow corrections of the results for any deviation from this assumption. Furthermore, data on twins can be augmented by collecting data on their family members as well as the familial environment, thereby providing information about how environmental factors interact with genetic predisposition to produce a disease.

Prior to 1992, twin studies firmly established substantial heritability of alcoholism in men but not in women. Since then, however, several studies have also reported a substantial heritability of alcoholism in women. For example, a study of adult Australian MZ and DZ twins suggested that about two-thirds of the risk of becoming alcoholic was genetically mediated in both men and women, with the remainder of the risk determined by environmental factors not shared by the two members of a given twin pair (Heath et al. 1997). The data provided no evidence for a difference in the degree of heritability in men and women, or for genetic factors operating in one gender but not the other. This last conclusion was particularly aided by analyses of data from opposite-sex twin pairs, a type of analysis not previously reported.

Because people who eventually become alcoholic typically begin experimenting with alcohol use during adolescence, investigators have long been interested in factors influencing initiation of alcohol use during adolescence. A study among Dutch families, each consisting of a pair of adolescent twins and their parents, indicated that children’s drinking behavior is influenced primarily by genetic and environmental factors other than their parents’ alcohol use (Koopmans and Boomsma 1996). This conclusion is consistent with findings from previous studies demonstrating strong peer influences on adolescent alcohol use.

Many alcoholics suffer from medical complications of alcoholism, such as liver cirrhosis, pancreatitis, cardiomyopathy, or psychosis due to brain damage. The inconsistency with which medical complications occur in alcoholism has led to the hypothesis that susceptibility to these complications is influenced by genetic factors independent of those influencing susceptibility to alcoholism itself. Researchers tested this hypothesis in male MZ and DZ twin pairs from the U.S. World War II Era Veteran Twin Registry, assessing the co-occurrence of alcoholism, cirrhosis, and alcoholic psychosis (Reed et al. 1996). From the MZ and DZ concordance rates for the three diseases, the investigators calculated heritabilities of 0.59 for alcoholism (in general agreement with results of other studies), 0.47 for liver cirrhosis, and 0.61 for alcoholic psychosis. For each trait, the remainder of the variance in susceptibility was due to environmental factors not shared by members of a twin pair. Using an analytic method that allowed for simultaneous analysis of all three diseases, the investigators calculated that 85 percent of the overall genetic risk was shared for alcoholism, cirrhosis, and psychosis.

Although many observers have noted that alcoholics smoke very heavily, the reasons for this dual substance use have been poorly understood. Recent twin studies are shedding considerable light on the reasons for this phenomenon. In one such study, researchers analyzed tobacco and alcohol use versus nonuse in adolescent and young adult Dutch twin pairs (Koopmans et al. 1997). At all ages tested (12 through 25 years), regular alcohol use was highly correlated with regular tobacco use. For 12- to 16-year-olds, shared environmental factors (peer pressure was very likely prominent among them) influenced both smoking and drinking. For 17- to 25-year-old men, however, both alcohol and tobacco use were highly genetically determined, with shared environmental influences playing a significant but lesser role. For 17- to 25-year-old women, alcohol use was highly genetically determined, and tobacco use was influenced by both genetic and shared environmental factors. Both in young adult men and women, the same genetic factors influenced alcohol as well as tobacco use. These findings suggest that while initial exposure to alcohol and nicotine is environmentally influenced, persistence in using these substances is under strong shared genetic influence.

The physiologic mechanism of the shared genetic influence on alcohol and tobacco consumption is currently a matter of speculation. One hypothesis is that individuals with high reactivity to stress may use both substances for stress relief. Alternatively, use of either substance may induce physiologic tolerance to the other, leading to a need to consume greater amounts of the latter substance in order to experience a subjective effect. Consistent with the latter hypothesis, several twin studies have suggested that smoking may increase the risk of alcoholism by reducing sensitivity to alcohol.

## Findings From Genetic Linkage Studies

Although twin and family studies can provide information about the genetic architecture of alcoholism and the relationship between genetic influences on alcoholism and other traits, they do not permit the identification of the specific genes influencing predisposition to alcoholism. Current efforts to identify such genes rely on genetic linkage and association studies. Such studies have received enormous impetus in recent years from the mapping of large numbers of human genetic markers and genes under the HGP and from the development of more sophisticated statistical methods for analyzing gene mapping data.

Genetic linkage studies can be designed in either of two principal ways. In the first design, investigators track the inheritance of the disease, along with that of genetic markers spanning the entire genome, through multigenerational families affected by the disease. In the second design, investigators measure the degree to which pairs of siblings (or other relatives) affected by the disease share different marker alleles. On average, simply by chance, siblings are expected to share half of the alleles of most of their genes. However, two siblings affected by the same disease will more frequently share alleles of markers close to genes affecting predisposition to, or progress of, the disease. Under either design, markers shown to be genetically linked to a disease define a chromosomal region likely to contain a gene influencing the disease. The advantage of this approach to gene discovery is that a sufficiently comprehensive marker map, such as that recently assembled by the HGP, permits an unbiased search of the entire genome without requiring any prior physiologic hypothesis about which genes might influence the disease.

The results of the first two systematic searches of the entire human genome (termed genome scans) for genes influencing predisposition to alcoholism have recently been published. The first study, by the Collaborative Study on Genetics of Alcoholism (COGA), a National Institute on Alcohol Abuse and Alcoholism-supported consortium of investigators at six centers across the United States, reported results from a primarily Caucasian-American sample of 987 individuals from 105 families (Reich et al. 1998). This study found suggestive evidence for genes influencing susceptibility to alcoholism on chromosomes 1 and 7 as well as weaker evidence for a gene on chromosome 2. It also reported modest evidence for a gene reducing the risk for alcoholism on chromosome 4. An independent genome scan, based on 152 subjects from a Southwestern American Indian tribe, reported suggestive evidence for a gene influencing susceptibility to alcoholism on chromosome 11 as well as suggestive evidence for a protective gene on chromosome 4 in approximately the same region implicated by the COGA study (Long et al. 1998).

It is not surprising that the two studies implicated different chromosomal regions because (1) American Indians and Caucasian Americans (of European descent) have different genetic histories, and (2) the physiologic mechanisms leading to alcoholism in American Indians may be different from those in Caucasians. In view of these differences between the two subject populations, it is of interest that both studies found some evidence for a protective gene in the same region of chromosome 4 (which includes, for example, the ADH2 and ADH3 genes).

## Findings From Genetic Association Studies

Genetic association tests measure whether a particular allele of a gene occurs more frequently in individuals affected by a disease than in unaffected individuals. A finding of genetic association can indicate that the gene under study influences the disease. Although such tests require prior knowledge of the gene under study (unlike genetic linkage tests), they are statistically much more powerful than linkage tests for detecting genes exerting only small effects on predisposition to a disease. They are also easier to perform than linkage tests, requiring ascertainment only of disease cases (and sometimes their parents) and controls rather than the entire nuclear families or large, multigenerational families required for linkage studies. However, since an apparent association between an allele and a disease can arise for reasons other than the influence of that allele on the disease, association studies need to be carefully designed.

A group of seven such well-designed studies focused on the association of the ADH2 and ADH3 genes with alcoholism. A meta-analysis of these studies, which were done in various ethnically matched Asian subject samples, attempted to measure to what extent genetic variation in these genes affects the risk of alcoholism (Reich et al. 1998). The analysis found consistent results across the seven studies, firmly establishing that certain alleles of these genes reduce the risk that their carriers will develop alcoholism.

Genes encoding various components of the nerve signal transmission system using the brain chemical (i.e., neurotransmitter) dopamine also have been tested for association with alcoholism. In contrast to early reports and despite some conflicting results, many well-designed studies found no evidence for the association of the dopamine receptor D2 with alcoholism. Some studies of the genes encoding other molecules in the dopamine pathway, however, have produced suggestive results indicating an association with alcoholism that is worthy of further pursuit. These genes include alleles of the tyrosine hydroxylase gene, which encodes an enzyme centrally involved in dopamine synthesis and functional variants of another dopamine receptor called D4.

These examples demonstrate that most genes tested so far for association with alcoholism have already been suspected of playing a role in predisposition to alcoholism. The power of genetic studies to reveal the influence of previously unsuspected genes on predisposition to alcoholism, thereby affording insights into previously unrecognized disease mechanisms, thus remains to be exploited, at least in genetic association studies.

## Psychosocial Factors in Alcohol Use and Alcoholism

No single, simple explanation exists for why some people develop problems with alcohol. Research has shown that multiple pathways involving psychosocial variables can lead to behaviors that involve alcohol consumption, ranging from simple alcohol experimentation to severe alcohol dependence. Furthermore, different subtypes of alcoholism exist that may have different causes, or etiologies. Finally, multiple biological and psychosocial factors mutually influence each other in causing alcohol abuse, and it would be incorrect to view psychosocial causes as either independent from, or competing with, biological causes. Rather, alcohol use and alcoholism are best viewed as end products of a combination of biopsychosocial influences.

This section reviews recent psychosocial research that has focused on four areas: family history of alcoholism, developmental issues, motivations, and alcohol-related cognitions (i.e., beliefs about alcohol).

## Family History of Alcoholism

A family history of alcoholism is a well-established risk factor for the development of alcoholism, although the majority of children of alcoholics (COAs) do not develop alcohol use disorders. COAs differ from children without a family history of alcoholism in several ways. These factors include a higher prevalence of mental and behavioral disorders, more adverse family environments, and physiologic responses to alcohol that are known to be associated with risk, in particular, a lack of sensitivity to alcohol’s intoxicating effects or an increased sensitivity to its anxiety-reducing effects. These characteristics are not unique to COAs, and the same factors that mediate risk of developing alcohol problems in children with a family history may also explain the risk faced by those without a family history. Models that seek to explain how these risk factors interact to lead to alcohol-related problems suggest that COAs are exposed to higher levels of these risk factors than are other children.

One source of the variation in the outcomes of COAs is that familial alcoholism occurs in different forms. Recent studies suggest that the type of alcoholic syndrome present in the family influences the child’s risk of having psychological characteristics associated with risk for alcoholism. Researchers have identified three subtypes of familial alcoholism risk (Finn et al. 1997):

- Familial alcoholism combined with low levels of other psychopathology
- High levels of both familial alcoholism and familial antisocial personality and violence
- High levels of familial alcoholism along with depression, mania, and anxiety disorders.

Predictably, young adult offspring from the families with alcoholism have elevated levels of alcohol problems compared with peers with no family history of alcoholism (Finn et al. 1997). In addition, offspring of the families with alcoholism and antisocial personality themselves have the highest levels of antisociality and negative affect (e.g., anxiety, depression, and neuroticism) compared with offspring of other alcoholic families.

Similar findings have emerged from a community sample of children preschool through age 8 (Zucker et al. 1996). In a comparison of children of families without alcoholism, families with alcoholism, and families with coexisting alcoholism and antisocial personality disorder, children whose families showed both alcoholism and antisociality had the highest levels of risk factors for developing alcohol problems and were also most likely to maintain this risk over time.

### Mediational Models

Recent investigations have attempted to understand the mechanisms or processes that underlie the effects of parental alcoholism on children. An important approach involves the development and testing of mediational models that provide an overall conception of how particular risk factors play out in the lives of the affected individuals. Three broad groups of theoretical models provide platforms for exploring the transmission of alcoholism from parent to child: “deviance proneness,” “negative affectivity” (or emotionality), and “sensitivity to the effects of alcohol.” The main characteristics of these models, which are not mutually exclusive, but are interrelated and interacting, are as follows:

The *deviance proneness model* focuses on deficits in children in behavioral self-regulation and socialization and on the cascade of effects that result from and interact with these deficits. According to this model, COAs have difficult temperaments and experience poor parenting, both of which place them at risk for emotional distress and failure in school. This, in turn, raises their risk for affiliation with a deviant peer group likely to promote alcohol use and misuse.

The *negative affectivity model* focuses on the importance of stress and negative affect in explaining the transmission of alcoholism from generation to generation. According to this model, COAs are exposed to high levels of life stress and are temperamentally hyper-reactive to stress. These children develop high levels of emotional distress and drink to relieve these feelings.

Models based on *sensitivity to alcohol’s effects* are founded on the hypothesis that COAs have greater sensitivity to alcohol’s stress response-dampening effects and less sensitivity to alcohol’s negative effects.

At least some experimental evidence exists for each of these models.

### The Role of Executive Functioning

Early conduct problems, which according to the deviance proneness model evolve into a broad set of undercontrolled behaviors (e.g., alcoholism), may be related to neuropsychological deficits in executive functioning. Executive functioning encompasses the capacity for sustained attention, concentration, abstract reasoning, goal setting, anticipation and planning, and the ability to monitor one’s own behavior (e.g., to inhibit inappropriate behavior and shift to adaptive behavior).

Poor executive functioning may predict increases in alcohol consumption among young adults with a family history of alcoholism. Several mechanisms may underlie the association of poor executive functioning with alcohol problems. For example, children with poor executive functioning are more difficult to parent, evoke more punishment, and thus may develop poorer bonds to parents and poorer socialization. Moreover, children with poor executive functioning are likely to experience more failure in school, which increases the risk of making friends with deviant peers and, subsequently, of escalating alcohol use in adolescence. Finally, people with deficits in executive function also may be unable to regulate their own mood, making them more sensitive to stress. These people would be particularly vulnerable to the stress response-dampening properties of alcohol.

### The Role of Parenting and the Family Environment

Researchers have examined parenting and family environment in an attempt to understand both the transmission of alcoholism from generation to generation and the causes of alcohol use and misuse in the wider population. In general, low levels of parental emotional support and a lack of control and monitoring of child behavior are linked to other adolescent problem behaviors, such as smoking and early sexual activity.

Some of the parenting deficits in alcoholic families are associated with the development of early conduct problems and early onset of alcohol use, which itself is a risk factor for later problems with alcohol use. Children of these families may not learn emotional and behavioral self-regulation and may lack social skills, which also increases the likelihood of rejection by mainstream peer groups and association with AOD-using peers. Such poor parenting and poor socialization may create a high risk of alcohol problems, not only for COAs, but also for adolescents from nonalcoholic families. However, poor parenting may be a product as well as a cause of behavioral difficulties in children. Thus, children with conduct disorders may evoke poor parenting responses.

### Protective Factors

Most COAs do not develop alcohol dependence. According to the mediational models described earlier, this would be partially due to these children not experiencing mediators of risk such as difficult temperaments or poor parenting. Alternatively, COAs subject to these risk mediators may have good outcomes because their risk is buffered by exposure to a protective factor.

Some recent evidence supports the existence of protective factors. One 3-year study of adolescents in alcoholic families found that these children were less likely to begin using AODs if they perceived that they had control over their environment, had good cognitive coping skills, and reported that their families were highly organized (Hussong and Chassin 1997). Other investigators have found that in alcoholic families that preserve family rituals, such as keeping to established daily routines and celebrating holidays, the young adult offspring are less likely to report problem drinking (Hawkins 1997). Furthermore, the National Longitudinal Study on Adolescent Health identified two factors that protected children from taking risks in four health areas, including AOD abuse (i.e., abuse of cigarettes, alcohol, and marijuana), emotional health, violence, and sexuality. These two protective factors were parent-family connectedness and school connectedness.^3^ Finally, researchers found that parental support had a protective effect, particularly with children’s mental health, apparently by enabling adolescents to cope better with life stresses, thereby preventing them from turning to heavy drinking.

## Developmental Issues

Alcohol use and alcoholism can also be studied within the context of psychosocial development throughout the life span. Early developmental antecedents to alcoholism can be seen even in the preschool years in the form of deficits in self-regulation, emotional reactivity, and conduct problems. For example, in one study, observers rated the temperaments of 3-year-old children; 18 years later the same individuals underwent diagnostic interviews (Caspi et al. 1996). Boys whose temperaments were rated as undercontrolled (i.e., impulsive, restless, and distractible) or inhibited (i.e., shy, fearful, and easily upset) at age 3 were more likely than other children to be diagnosed at age 21 as alcohol dependent or as having alcohol-related problems.

Developmental researchers also look at age-related peaks and declines in alcohol use. Drinking usually begins in adolescence, and early initiation of alcohol use is a risk factor for alcohol-related problems in adulthood. In general, the factors that predict alcohol involvement among adolescents are similar to those that predict other forms of adolescent problem behavior, such as delinquency and risky sexual behavior. These predictors include high life stress, nonadaptive coping styles, parental and peer AOD use, little parental support, a low level of academic competence, and poor behavioral control.

In older adolescents and young adults, developmental changes related to their new freedoms and responsibilities influence their drinking behavior. For example, when young adults take on the responsibilities of work and marriage, they typically reduce their drinking and are less likely to report symptoms of alcohol abuse and dependence. One interpretation is that these individuals drink less during this period because drinking is incompatible with the obligations of adult roles. These findings are consistent with past research indicating that a subtype of alcoholism may be developmentally limited; that is, some people may drink heavily and have alcohol-related problems in young adulthood but not in later years. Indeed, investigators are finding more evidence to support the idea that different subtypes of alcoholism start at different ages, and have different causes. Establishing such classification schemes for alcoholics is not an abstract pursuit because the treatment needs of these groups likely differ.

## Motivation To Drink

One area of psychosocial research on alcohol use focuses specifically on what motivates individuals to drink. Perhaps the most commonly studied motivation involves alcohol’s ability to reduce anxiety, thus making it a way to cope with stress. The strength of the relationship between stress and alcohol consumption may vary across the life span, however, being weaker in adolescents and more pronounced in older adults.

### Stress Reduction

Some people use alcohol to cope with stress. One model proposes that negative emotions (e.g., anxiety or depression), the expectation that alcohol will relieve these feelings, and coping styles characterized by avoiding rather than confronting life issues all may increase a person’s motivation to drink in order to cope with stress. Consistent with this model, these characteristics show the strongest correlation between stress and drinking. The evidence that some people use alcohol to reduce stress, however, is complex and inconsistent for a number of reasons, not least of which is that there are multiple determinants of alcohol use. Furthermore, the effect of protective factors that reduce the impact of stress on drinking (e.g., social support systems) complicates the evidence for the relationship. Finally, problems such as a time lag between the occurrence of a stressful event and resulting alcohol use also may result in inconsistent findings. Thus, one study using daily diaries found that women consumed less alcohol in high-stress weeks (perhaps because alcohol impaired their ability to cope with stressors) but consumed more alcohol after the stressful event was over (Breslin et al. 1995).

### Mood Enhancement

Other motives and determinants of alcohol use can overshadow stress-reduction motives. One model suggests that people who are characterized by high levels of sensation seeking or who expect that alcohol use will enhance positive mood will be more strongly motivated to drink for this effect (Cooper et al. 1995). Alcohol use to reduce stress or enhance positive mood are not mutually exclusive motivations to drink, however, and they can be observed in the same person. The most severe alcohol problems have been reported in people who are characterized by both high levels of negative affect and low levels of constraint.

### Alcohol’s Effect on Emotional State

Questions remain as to how exactly alcohol affects emotional state. Laboratory data show that alcohol dampens responses to stress; at the same time, however, alcohol can increase anxiety in some people. Recently, investigators attempted to determine whether alcohol produced a specific decrease in negative affect or whether it simply reduced emotional arousal across the board, muting the intensity of any emotion (Stritzke et al. 1995). The study suggested that alcohol generally reduces emotional arousal, rather than specifically diminishing responses occurring during positive emotional states. If the effect of alcohol consumption is generally to lower emotional arousal, however, then it is unclear how alcohol acts to enhance emotional state. Investigators have suggested that alcohol’s effects on emotional reactivity may result from its effects on cognition and information processing, rather than on motivational systems involving affect and emotion.

### External Motivations To Drink

Social influences, norms, and contexts also play a role in the motivation to drink. External motives to drink include the social rewards of projecting a particular image, as well as the avoidance of social rejection by complying with perceived social norms that include consuming alcohol in social settings.

## The Role of Cognition: Beliefs About Alcohol

Researchers also have investigated alcohol-related cognition (i.e., the conscious and unconscious knowledge or beliefs about alcohol) and the role of these beliefs in shaping alcohol-related behavior. Based on direct experience with the pharmacologic effects of alcohol and vicarious learning from parents, peers, and the broader culture and media, people develop expectancies about the effects of alcohol consumption on their behavior. These expectancies then influence their decisions to drink. Theorists have suggested that cognition may in some cases be a bridge between the primary reinforcing effects of alcohol and individual decisions to use it in a particular situation.

### Explicit Beliefs and Expectations

Most people can describe many of their beliefs and expectations about alcohol. These beliefs are conscious or explicit. Expectations about alcohol’s effects begin developing early in life, even before a person drinks any alcohol, and predict future alcohol use. For example, young adolescents who told researchers that they believed alcohol makes it easier to socialize were shown in later years to have increased their drinking over time to higher rates than did their peers without this belief (Smith et al. 1995). Data also suggest that expectancies and the experience of drinking have reciprocal effects. Not only do expectancies predict later drinking but drinking experiences shape later expectancies about alcohol’s effects.

### Implicit Beliefs and Expectations

Researchers also have attempted to identify the alcohol-related beliefs, memory associations, and emotional states that are activated more spontaneously, without conscious awareness— termed “implicit cognition”—and to study their role in drinking behavior. Such studies have measured associative memory processes in diverse ways, including investigating how people at various ages mentally organize associations between alcohol and its effects, measuring free associations to alcohol-related words and pictures, and observing how exposure to an alcohol-related concept affects a participant’s response to later stimuli.

Little is yet known about the relationship between implicit and explicit beliefs about alcohol and the potential differences in the way that the two types of knowledge influence alcohol use. One hypothesis suggests that conscious, explicit expectations influence alcohol use through deliberate, conscious decisionmaking. In contrast, unconscious memory associations may influence alcohol use more spontaneously when the expectations are triggered in an immediate situation.

## Conclusions

The ultimate goal of genetic analyses, such as QTL studies, in alcohol research is identifying the genes that contribute to the development of alcoholism. Analysis of these genes would allow a rapid exploration of the biochemical underpinnings of alcohol’s actions and would link behavioral change to underlying genetic predisposition and biochemical action. Although no equivalent to human alcoholism exists in animals, genes identified in animal models almost certainly have human equivalents that are also involved in alcohol’s actions and that may predispose to human alcoholism. Such genes and the proteins they encode are potent targets for intervention, both diagnostic and pharmacologic. It seems certain that the results of genetic analyses will be exploited dramatically in the next century to provide a variety of “designer drugs,” perhaps targeted to individual problems associated with particular forms of alcohol abuse. Because mapping the genes that influence genetically complex diseases presents difficult challenges for investigators progress on these diseases has been much slower than progress in gene mapping for single-gene disorders.

Twin studies, which explore the relationship between alcoholism and other traits, continue to contribute to the formulation of a more biologically valid definition of the disease and to the characterization of disease subtypes that may ultimately prove to have differing genetic bases. Achievement of these objectives would greatly expedite the gene search. Further progress toward the precise identification of genes influencing predisposition to alcoholism will depend on the development of improved tools for the gene-discovery enterprise. Foremost among these tools will be more sophisticated statistical methods, a complete human gene map, and a catalogue of the major human genetic variations. Once genes influencing the predisposition to alcoholism have been identified, a major new challenge confronting genetic epidemiologists will be to understand how such genes interact with environmental factors to influence the development of alcoholism in the general population.

Research on psychosocial factors in alcohol consumption and alcoholism encompasses a broad range of investigations, all aimed at understanding how multiple biological and psychosocial risk factors interact to influence alcohol-related behavior. Research on familial transmission of alcoholism in particular focuses on how genetic vulnerabilities are translated in the context of the family and social environment into alcoholism.

Recent research traces the evolution of the disorder of alcoholism along the life span and teases out the emotional and cognitive motivational factors that induce people to drink. By constructing models of how the risk factors identified interact and then testing these models empirically, scientists are identifying risk factors for alcohol misuse as well as potential mediators and moderators of this risk. The ultimate goal of this research is to develop preventive interventions that target these risk and protective factors in order to reduce the prevalence of alcohol-related illness and death.

## Footnotes

1 For a definition of this and other technical terms used in this article, see the glossary, p. 25.

2 In June 2000 HGP researchers, together with researchers from the biotechnology company Celera, announced that they had determined the DNA sequence and completed the first map of the human genome.

3 Children who experienced parent-family connectedness said they felt close to their mother and/or father, perceived that either or both of their parents cared about them, expressed satisfaction with their relationship with either or both of their parents, and felt loved and wanted by family members. School connectedness was experienced as a feeling of being treated fairly by teachers, being close to people at school, and being part of one’s school.

## GLOSSARY

Alcohol dehydrogenase (ADH): An enzyme involved in alcohol metabolism; converts alcohol into acetaldehyde.
Aldehyde dehydrogenase (ALDH): An enzyme involved in alcohol metabolism; converts acetaldehyde into acetic acid.
Allele: One of two or more variants of a gene that occupy the same location on a *chromosome.*
Chromosomes: Small, threadlike structures in the cell nucleus that contain the genetic blueprint of an organism. Humans have 46 chromosomes in each cell.
Dopamine: A brain chemical (i.e., neurotransmitter) that mediates the rewarding properties of alcohol and other drugs.
Genome: The entire genetic material of an organism.
Inbred strains: Animal strains that have been generated by repeated inbreeding (e.g., brother-sister matings) and in which all the animals have identical genetic material (i.e., fixed allelic status).
Mapping: Determining the position of a gene relative to existing *markers* on a *chromosome*.
Marker: A DNA sequence whose position on a specific *chromosome* is known.
Phenotype: An observable characteristic, or trait, of an organism.
Quantitative trait: A complex trait, or behavior, that is determined by multiple genes and which is expressed along a spectrum from high to low.
Quantitative trait locus (QTL): A short stretch of DNA that contains a gene contributing to a multigenetic, or *quantitative, trait*.
QTL mapping: A technique used to search for genes that influence multigenetic, or *quantitative, traits* (i.e., complex behaviors determined by multiple genes). This approach is based on the concept of linkage, which posits that genes located close together on the *chromosome* are more likely to be inherited together from one parent than two genes further apart. This technique provides a means of locating and measuring the effects of a single *quantitative trait locus* (QTL) on a trait (i.e., *phenotype*).
Receptor: A protein located on the cell surface that serves as a “docking molecule” for signaling molecules, such as neurotransmitters and hormones.
Recombinant inbred (RI) strains: Animal strains generated by mating two *inbred strains* and then inbreeding the F2 (“grandchild”) generation; in these RI strains, the genetic material from the original inbred strains has been recombined as a result of the DNA rearrangement that occurs during the specialized cell division (i.e., meiosis) that results in the production of egg and sperm cells.
Stress-response dampening: An effect of alcohol that results in a moderation of the drinker’s physiological response to stressful situations.

## References

[b1-arcr-24-1-17] Breslin FC, O’Keefe MK, Burrell L, Ratliff-Crain J, Baum A (1995). The effects of stress and coping on daily alcohol use in women. Addictive Behavior.

[b2-arcr-24-1-17] Buck KJ, Metten P, Belknap JK, Crabbe JC (1997). Quantitative trait loci involved in genetic predisposition to acute alcohol withdrawal in mice. Journal of Neuroscience.

[b3-arcr-24-1-17] Caspi A, Moffitt TE, Newman DL, Silva PA (1996). Behavioral observations at age 3 years predict adult psychiatric disorders: Longitudinal evidence from a birth cohort. Archives of General Psychiatry.

[b4-arcr-24-1-17] Cooper ML, Frone M, Russell M, Mudar P (1995). Drinking to regulate positive and negative emotions: A motivational model of alcohol use. Journal of Personal and Social Psychology.

[b5-arcr-24-1-17] Crabbe JC, Phillips TJ, Gallaher EJ, Crawshaw LI, Mitchell SR (1996). Common genetic determinants of the ataxic and hypothermic effects of ethanol in BXC/Ty recombinant inbred mice: Genetic correlations and quantitative trait loci. Journal of Pharmacology and Experimental Therapy.

[b6-arcr-24-1-17] Erwin VG, Markel PD, Johnson TE, Gehle VM, Jones BC (1997). Common quantitative trait loci for alcohol-related behaviors and CNS neurotensin measures. Hypnotic and hypothermic effects. Journal of Pharmacology and Experimental Therapy.

[b7-arcr-24-1-17] Finn PR, Sharkansky EJ, Viken R, West TL, Sandy J, Bufferd GM (1997). Heterogeneity in the families of sons of alcoholics: The impact of familial vulnerability type on offspring characteristics. Journal of Abnormal Psychology.

[b8-arcr-24-1-17] Hawkins CA (1997). Disruption of family rituals as a mediator of the relationship between parental drinking and adult adjustment in offspring. Addictive Behaviors.

[b9-arcr-24-1-17] Heath AC, Bucholz KK, Madden PA, Dinwiddie SH, Slutske WS, Bierut LJ, Statham DJ, Dunne MP, Whitfield JB, Martin NG (1997). Genetic and environmental contributions to alcohol dependence risk in a national twin sample: Consistency of findings in women and men. Psychology and Medicine.

[b10-arcr-24-1-17] Hussong AM, Chassin L (1997). Substance use initiation among adolescent children of alcoholics: Testing protective factors. Journal of Studies on Alcohol.

[b11-arcr-24-1-17] Koopmans JR, Boomsma DI (1996). Familial resemblance in alcohol use: Genetic or cultural transmission?. Journal of Studies on Alcohol.

[b12-arcr-24-1-17] Koopmans JR, van Doornen LJ, Boomsma DI (1997). Association between alcohol use and smoking in adolescent and young adult twins: A bivariate genetic analysis. Alcoholism: Clinical and Experimental Research.

[b13-arcr-24-1-17] Long JC, Knowler WC, Hanson RL, Robin RW, Urbanek M, Moore E, Bennett PH, Goldman G (1998). Evidence for genetic linkage to alcohol dependence on chromosomes 4 and 11 from an autosome-wide scan in an American Indian population. American Journal of Medicine and Genetics.

[b14-arcr-24-1-17] Melo JA, Shendure J, Pociask K, Silver LM (1996). Identification of sex-specific quantitative trait loci controlling alcohol preference in C57BL/6 mice. Nature Genetics.

[b15-arcr-24-1-17] Phillips TJ, Lessov CN, Harland RD, Mitchell SR (1996). Evaluation of potential genetic associations between ethanol tolerance and sensitization in BXC/Ty recombinant inbred mice. Journal of Pharmacology and Experimental Therapy.

[b16-arcr-24-1-17] Reed T, Page WF, Viken RJ, Christian JC (1996). Genetic predisposition to organ-specific endpoints of alcoholism. Alcoholism: Clinical and Experimental Research.

[b17-arcr-24-1-17] Reich T, Edenberg HJ, Goate A, Williams JT, Rice JP, Van Eerdewegh P, Foroud T, Hesselbrock V, Schuckit MA, Bucholz K, Projesz B, Li TK, Conneally PM, Nurnberger JI, Tischfield JA, Crowe RA, Cloninger CR, Wu W, Shears S, Carr K, Crose C, Willig C, Begleiter H (1998). Genome-wide search for genes affecting the risk for alcohol dependence. American Journal of Medicine and Genetics.

[b18-arcr-24-1-17] Smith GT, Goldman MS, Greenbaum PE, Christiansen BA (1995). Expectancy for social facilitation from drinking: The divergent paths of high-expectancy and low-expectancy adolescents. Journal of Abnormal Psychology.

[b19-arcr-24-1-17] Stritzke WG, Patrick CJ, Lang AR (1995). Alcohol and human emotion: A multidimensional analysis incorporating startle probe methodology. Journal of Abnormal Psychology.

[b20-arcr-24-1-17] Zucker RA, Ellis DA, Bingham CR, Fitzgerald HE (1996). The development of alcoholic subtypes: Risk variation among alcoholic families during the childhood years. Alcohol Health & Research World.

